# Varicella-zoster virus infection: a review about varicella and herpes zoster^☆^

**DOI:** 10.1016/j.abd.2026.501367

**Published:** 2026-05-09

**Authors:** Maria Paula Barbieri D'Elia, Carla Riama Lopes de Pádua Moura, Rafael de Deus Moura, Juliana de Sá Pires Carvalho, Henrique Pott

**Affiliations:** a Postgraduate Gerontology Program, Universidade Federal de São Carlos, São Carlos, SP, Brazil; b Vigilare Lab, Department of Medicine, Universidade Federal de São Carlos, São Carlos, SP, Brazil; c University Hospital, Universidade Federal do Piauí, Teresina, PI, Brazil; d Department of Specialized Medicine, Universidade Federal do Piauí, Teresina, PI, Brazil

**Keywords:** Chickenpox, Chickenpox vaccine, Herpes zoster, Herpes zoster vaccine, Varicella zoster virus infection

## Abstract

The Varicella-zoster virus (VZV) is a prevalent human pathogen that links dermatology, virology, and immunology. Following primary varicella infection, the virus establishes lifelong latency in the sensory ganglia, with reactivation manifesting as herpes zoster. The clinical spectrum ranges from typical dermatomal vesicular eruptions to atypical or disseminated presentations in older adults or immunocompromised patients, often posing diagnostic challenges. Early recognition and prompt initiation of antiviral therapy are essential to limit lesion progression, reduce viral shedding, and prevent complications. Dermatologists are uniquely positioned to identify these manifestations and distinguish VZV infection from its clinical mimics. Advances in molecular diagnostics have improved the detection of atypical cases, while the introduction of the attenuated-virus vaccine for varicella and recombinant glycoprotein E–based vaccine for herpes zoster has transformed prevention, providing durable protection even in older adults and immunosuppressed populations. Beyond therapy, dermatologists play a key role in integrating vaccination assessment and patient education into routine care. Understanding the biological continuum of VZV (from latency to reactivation) enhances diagnostic precision, guides evidence-based treatment, and supports immunization strategies against VZV. As VZV continues to impose a substantial burden of cutaneous and neuropathic morbidity, an integrated dermatological approach that combines early therapeutic intervention with preventive counseling represents the most effective strategy to reduce its clinical and public health impact.

## Introduction

The Varicella-zoster virus (VZV, human herpesvirus 3) is a ubiquitous human pathogen of the *Herpesviridae* family responsible for two clinically distinct conditions: primary infection or varicella (chickenpox) and viral reactivation, known as herpes zoster (shingles).^1^, ^2^ Both entities are highly relevant in dermatological practice, not only because of their characteristic cutaneous patterns but also because of their potential complications, including chronic pain syndromes, bacterial superinfection, and postherpetic neuralgia.^3^, ^4^

Although vaccination programs have reshaped the epidemiological landscape, VZV remains a substantial health concern, particularly in adults, older patients, and immunocompromised hosts.^3^, ^5^ Therefore, understanding its virology, immunopathogenesis, and clinical behavior is essential for dermatologists, who are often the first to recognize the disease and initiate timely antiviral therapy.

This review provides an updated, clinically oriented synthesis of VZV-related skin diseases tailored for dermatology practitioners. It integrates the fundamental aspects of viral biology, epidemiology, and host interactions with practical guidance on diagnosis, management, and prevention. Emphasis is placed on clinical reasoning and decision-making, translating current scientific knowledge into evidence-based dermatologic care practices.

## Essential virology and pathogenesis

VZV is an enveloped, double-stranded DNA virus that belongs to the *Alphaherpesvirinae* subfamily.^2^, ^6^ Its genome encodes more than 70 proteins, including structural and regulatory components essential for viral replication, latency, and immune evasion.^6^, ^7^ Although VZV shares structural similarities with Herpes Simplex Viruses (HSV-1 and HSV-2), it exhibits unique biological behaviors and tissue tropism, with a strong predilection for cutaneous and neuronal cells.^8^

During primary infection, VZV enters the host through the respiratory mucosa or conjunctiva and replicates in the regional lymph nodes.^2^ Transient viremia subsequently disseminates the virus to the skin, leading to the formation of characteristic vesicular lesions.^2^ Although these lesions contain high viral loads, transmission occurs predominantly via the respiratory route through inhalation of virus-containing aerosols and droplets.^2^ Following resolution of the primary infection, VZV establishes lifelong latency in the sensory dorsal root and cranial nerve ganglia, where it persists in a non-replicative state.^9^, ^10^ Unlike HSV, which may reactivate repeatedly, VZV typically remains dormant for decades until reactivation is triggered by waning cell-mediated immunity, most often in older adults and immunocompromised patients.^11^ The reactivated virus travels centrifugally along the sensory nerves to the skin, giving rise to a localized vesicular eruption of herpes zoster.^8^

The viral genome encodes several glycoproteins critical for infection and immune evasion, among which glycoprotein E (gE) is the most abundant and primary target of neutralizing antibodies.^2^ Viral replication predominantly occurs in the nuclei of epithelial and neuronal cells, inducing cytopathic changes that underlie the vesicular morphology of lesions.^2^

The pathogenesis is driven by intricate interactions between the virus and the host. During primary infection, humoral immunity contributes to viral clearance; however, long-term control and latency depend on cellular immunity, particularly VZV-specific CD4+ and CD8+ T-lymphocytes.^2^, ^11^ With advancing age or under immunosuppressive conditions, T-cell-mediated surveillance declines, facilitating viral reactivation.^2^, ^11^ The ensuing inflammation of the affected ganglia and nerves produces both cutaneous manifestations and neuropathic pain, which define herpes zoster.^9^, ^12^

Latency is maintained through the suppression of lytic gene expression and modulation of neuronal survival pathways, whereas reactivation induces neuronal damage that may persist beyond lesion resolution, manifesting as postherpetic neuralgia.^9^, ^11^, ^12^ The clinical diversity of VZV infections (including atypical, multidermatomal, and disseminated forms) stems from the balance between viral replication and host immune competence.^9^

## Epidemiology and clinical relevance

VZV infection is nearly universal in unvaccinated populations, with most individuals acquiring primary varicella infections during childhood.^13^, ^14^ In temperate climates, varicella typically occurs in early childhood, whereas in tropical regions, the infection may be delayed until adolescence or adulthood.^14^ Before the introduction of routine vaccination, varicella caused substantial morbidity, particularly among adults, pregnant women, and immunocompromised patients.^5^

Following widespread immunization with the live-attenuated varicella vaccine, the incidence of primary infection has declined substantially, resulting in reduced circulation of the wild-type virus within the community.^13^ This epidemiological shift has been proposed as a contributing factor to changes in herpes zoster incidence, partly due to diminished opportunities for exogenous immune boosting.^15^ However, long-term population-based analyses from the United States have not demonstrated the pronounced post-vaccination surge in herpes zoster incidence predicted by early models, suggesting a more complex and context-dependent relationship.^15^, ^16^ Despite decreased transmission, VZV remains endemic, sustained through lifelong latency in previously infected individuals.^11^ Consequently, herpes zoster continues to represent a significant public health burden, as viral reactivation may occur decades after the initial infection.^4^, ^17^, ^18^

The lifetime risk of developing herpes zoster in immunocompetent individuals is estimated to be approximately one in three, increasing sharply with age.^17^, ^19^ This reflects the progressive decline in VZV-specific cell-mediated immunity that accompanies immunosenescence.^20^, ^21^, ^22^ Additional risk factors include immunosuppressive therapy, malignancies, HIV infection, and chronic diseases such as diabetes mellitus and renal failure.^23^

From a dermatological perspective, VZV reactivation is clinically significant, not only for its cutaneous manifestations but also for its systemic impact.^24^, ^25^ Complications such as bacterial superinfection, postherpetic neuralgia, and ophthalmic or neurological involvement can result in long-term morbidity and a diminished quality of life.^24^, ^26^ The introduction of recombinant zoster vaccines has substantially improved prevention in older adults; however, coverage and adherence remain inconsistent worldwide.^27^, ^28^, ^29^, ^30^ For dermatologists, understanding the epidemiological landscape of VZV is crucial for anticipating disease patterns, identifying at-risk populations, and promoting preventive vaccination.

## Clinical manifestations

The dermatological spectrum of VZV infection encompasses two clinically distinct entities: primary varicella and herpes zoster.^2^, ^11^ Despite their differing epidemiological profiles, both share the same viral etiology and pathogenic foundation.^1^, ^2^, ^8^

### Varicella (primary infection)

**Figure 1:**
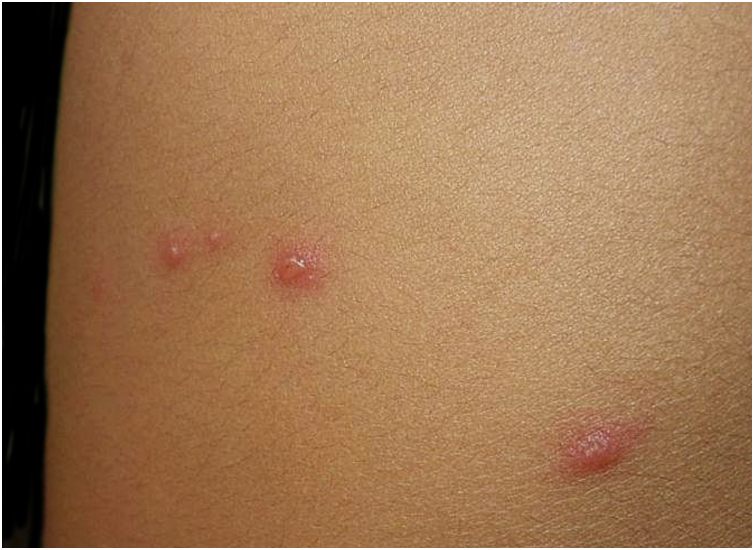
Acute varicella lesion. Courtesy: Lauro Lourival Lopes Filho, MD, Ph.D.

**Figure 2:**
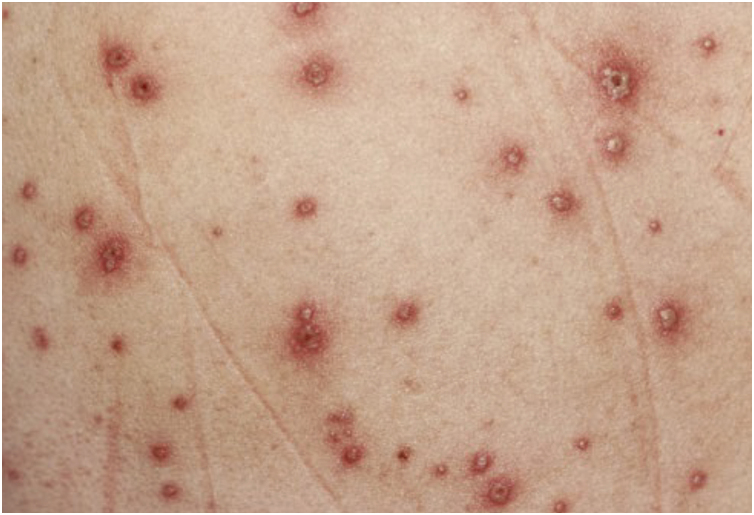
Polymorphic rash of varicella. Courtesy: Silvio Alencar Marques, MD, PhD.

Primary infection with VZV results in varicella or chickenpox, a generalized vesicular exanthem typically preceded by a brief prodromal phase characterized by low-grade fever, malaise, and mild respiratory symptoms.^31^, ^32^, ^33^ The eruption classically follows a craniocaudal progression and manifests as successive crops of pruritic vesicles on an erythematous base, often described as a “dew drop on a rose petal” (Fig. 1).^31^, ^34^ These lesions evolve asynchronously into pustules and crusts, and consequently, lesions at different stages of development commonly coexist within the same anatomical area (Fig. 2).^31^, ^35^ The exanthem predominates on the trunk and face, with relative sparing of the extremities.^31^ In addition to cutaneous involvement, vesicles or erosions of the oral mucosa are frequent, and conjunctival involvement may also occur, although less commonly.^31^, ^35^

In healthy children, varicella is usually self-limiting.^33^, ^35^ However, in adults, pregnant women, and immunocompromised patients, the disease can be severe, with complications such as pneumonia, hepatitis, and encephalitis.^31^, ^33^, ^35^ Secondary bacterial infections of excoriated lesions are common, particularly in young children.^31^, ^33^, ^35^ Cutaneous scarring may occur in severe or hemorrhagic forms of the disease, which is rare.^31^

### Herpes zoster (reactivation)

**Figure 3:**
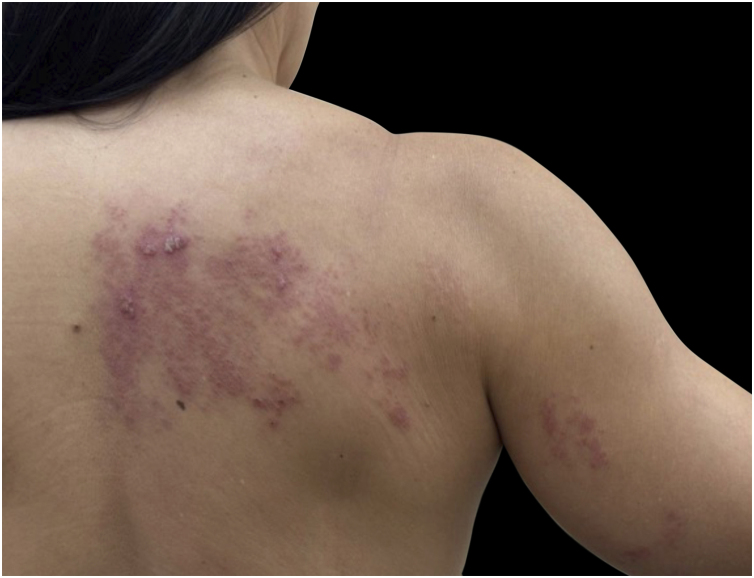
Herpes zoster: typical dermatomal involvement with erythema and vesicles.

Herpes zoster, or shingles, represents reactivation of latent VZV from sensory ganglia.^9^, ^36^, ^37^ The condition typically begins with localized pain, burning, or paresthesia along a dermatomal distribution, followed by the appearance of grouped vesicles on an erythematous base.^37^ The eruption is unilateral and does not cross the midline, providing a key diagnostic clue.^9^, ^37^ The thoracic and cranial dermatomes are the most frequently involved (Fig. 3).^9^, ^24^, ^38^

In selected patients (particularly those who are immunocompromised), VZV reactivation may be accompanied by transient viremia, leading to the appearance of vesicular lesions at sites distant from the primary dermatome.^39^, ^40^ In such cases, disseminated herpes zoster may partially mimic primary varicella, with scattered varicella-like lesions coexisting with a dominant unilateral dermatomal eruption.^39^, ^40^, ^41^ This atypical presentation can complicate clinical recognition and management, underscoring the importance of careful assessment of lesion distribution and host immune status.

Lesions typically evolve through vesicular, pustular, and crusting stages over 7–10 days.^9^ New vesicles may continue to emerge for several days, and pain frequently precedes the appearance of the rash or persists beyond its resolution.^9^ In a subset of patients (particularly older adults), postherpetic neuralgia may endure for months or even years.^12^, ^42^

**Figure 4:**
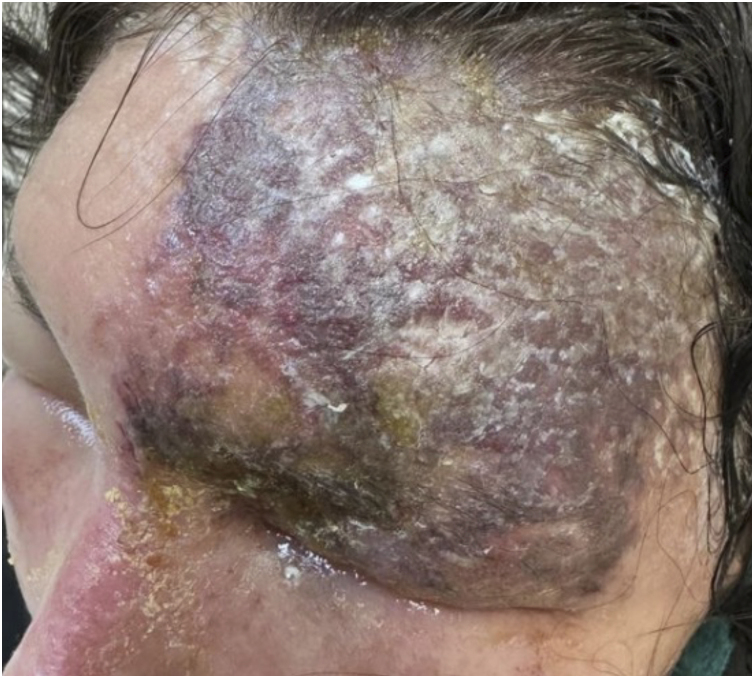
Extensive necrotic herpes zoster in an HIV-infected patient. Severe unilateral dermatomal involvement with extensive vesiculation and necrotic changes. Courtesy: Hiram Larangeira de Almeida Jr., MD, PhD.

Atypical presentations are relatively common and may obscure diagnosis. Herpes zoster *sine herpete* presents as dermatomal pain in the absence of cutaneous lesions, whereas disseminated zoster can mimic primary varicella infection, particularly in immunocompromised individuals.^10^, ^11^, ^43^ In this context, herpes zoster may manifest as exuberant and destructive morphologies, including extensive vesiculation with necrotic changes. Fig. 4 illustrates a severe necrotic presentation in an HIV-infected patient, in which, despite marked tissue damage, lesions remained strictly confined to a single dermatomal distribution, reinforcing unilateral involvement as a critical diagnostic feature, even in advanced or atypical cases.^23^, ^44^

**Figure 5:**
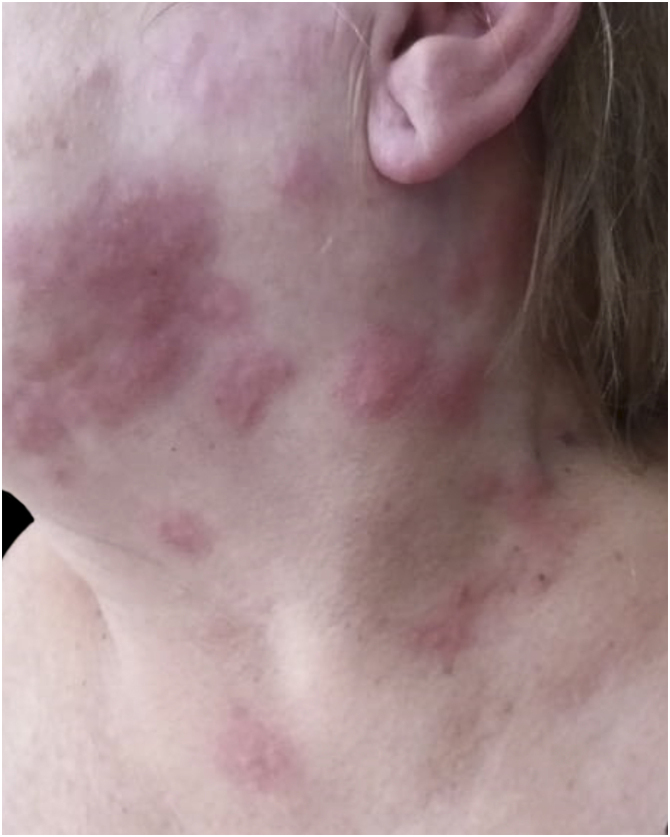
Cervical herpes zoster. Grouped vesicles on an erythematous base were distributed unilaterally along a cervical dermatome, respecting the midline. Courtesy: Hiram Larangeira de Almeida Jr., MD, PhD.

**Figure 6:**
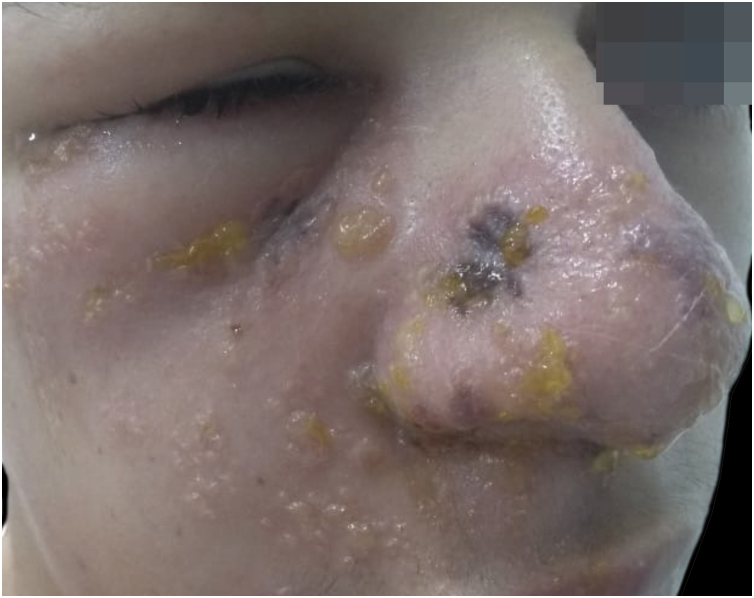
Facial herpes zoster. Unilateral vesicular eruption involving the facial region, consistent with the trigeminal nerve distribution.

The involvement of anatomically complex regions, such as the cervical and facial areas, can complicate diagnosis.^10^, ^45^ Cervical and facial herpes zoster may resemble other inflammatory, vesiculobullous, or infectious dermatoses, especially early on (Figure 5, Figure 6).^2^, ^9^, ^10^, ^45^ In both cases, recognition of a unilateral dermatomal pattern that respects the midline is essential for accurate diagnosis.^36^

Reactivation of the trigeminal ganglion, on the face, may lead to ophthalmic complications, including keratitis, uveitis, and permanent visual impairment.^3^, ^23^, ^45^, ^46^ Ramsay Hunt syndrome, resulting from facial nerve involvement, is characterized by vesicular lesions affecting the external ear or oral mucosa and is frequently accompanied by facial paralysis and auditory symptoms.^3^, ^23^, ^45^

In dermatological practice, prompt recognition of early, atypical, and anatomically challenging presentations is essential. Early initiation of antiviral therapy limits viral replication, shortens the duration of acute pain, and reduces the risk of cutaneous and neurological complications.^47^, ^48^, ^49^

## Complications

The complications of VZV infection range from mild cutaneous sequelae to severe systemic disease, depending on host immunity and age.^3^, ^45^, ^50^ Although most cases resolve uneventfully, the burden of complications (particularly those associated with herpes zoster) remains substantial and clinically relevant for dermatologists.

### Cutaneous complications

The most frequent cutaneous complication is a secondary bacterial infection of excoriated or ruptured vesicles, commonly caused by *Staphylococcus aureus* or *Streptococcus pyogenes.*^3^, ^5^ Impetiginization can result in scarring or post-inflammatory dyspigmentation.^33^, ^35^ In rare instances, necrotizing fasciitis may develop, particularly in immunocompromised patients.^3^, ^5^ Chronic or ulcerative lesions are also observed in patients receiving long-term corticosteroid or other immunosuppressant therapy.^3^, ^5^, ^23^

### Neurologic complications

Postherpetic Neuralgia (PHN) is the most debilitating sequelae of herpes zoster.^51^ It is characterized by persistent neuropathic pain lasting for at least 90-days after rash onset.^52^ The incidence and severity of PHN increase with age, reflecting cumulative neuronal injury and reduced regenerative capacity of the nervous system.^53^ Pathophysiologically, PHN results from inflammation, demyelination, and necrosis of the affected sensory neurons, leading to altered pain signaling within the dorsal horn.^54^, ^55^, ^56^ Clinically, patients describe burning, shooting, or stabbing pain, often accompanied by allodynia or hyperesthesia.^50^, ^52^

Other rare neurological complications include meningoencephalitis, transverse myelitis, and cranial nerve paralysis.^3^, ^5^ VZV vasculopathy, resulting from viral invasion of the cerebral arteries, can manifest as stroke or focal neurological deficits.^3^, ^5^, ^9^ In immunocompromised individuals, disseminated infections may involve the central nervous system, with high morbidity.^23^, ^44^, ^57^

### Ophthalmic and otologic complications

Herpes zoster *ophthalmicus*, arising from the reactivation of the ophthalmic branch of the trigeminal nerve, can cause keratitis, uveitis, and retinitis, potentially leading to permanent vision loss.^46^, ^58^ The presence of vesicular lesions on the tip of the nose (Hutchinson’s sign) indicates nasociliary nerve involvement and predicts ocular complications.^46^, ^58^ Ramsay Hunt syndrome, caused by involvement of the geniculate ganglion, presents with auricular vesicles, facial paralysis, and sensorineural hearing loss, often requiring multidisciplinary care.^58^, ^59^

### Systemic complications

Systemic dissemination of VZV can lead to serious extracutaneous complications, including pneumonitis, hepatitis, and disseminated intravascular coagulation.^33^, ^35^, ^45^ These events occur predominantly in immunocompromised individuals and during pregnancy, in whom viral replication is often more extensive and immune control is diminished.^23^, ^60^ Varicella pneumonia represents the most severe of these manifestations, carrying a mortality rate of 10%‒30% in untreated adults and requiring prompt recognition to avoid rapid respiratory deterioration.^33^, ^35^

Transplacental transmission during pregnancy may result in congenital varicella syndrome, a rare but devastating condition marked by limb hypoplasia, cicatricial skin lesions, ocular abnormalities, and neurodevelopmental impairment.^35^, ^60^ Additional risks emerge when aspirin is administered during active varicella infection, given its association with Reye syndrome, characterized by acute encephalopathy and hepatic steatosis.^35^

For dermatologists, maintaining a high index of suspicion regarding these systemic outcomes is essential. Early identification of cutaneous patterns suggestive of severe or disseminated disease supports timely referral, multidisciplinary coordination, and initiation of appropriate antiviral therapy, thereby reducing the likelihood of long-term morbidity.

## Diagnosis

The diagnosis of VZV infection is primarily clinical and is supported by the characteristic morphology and distribution of skin lesions. However, atypical presentations, immunocompromised hosts, and early or late disease stages may require laboratory confirmation of diagnosis.

### Clinical diagnosis

Pattern recognition remains central to the clinical diagnosis of varicella-zoster virus infections.^2^, ^11^ Varicella typically presents with lesions at different stages of evolution (macules, papules, vesicles, and crusts) distributed in a centripetal pattern involving the trunk, face, and scalp.^33^, ^35^ In contrast, herpes zoster produces a unilateral dermatomal eruption of grouped vesicles on an erythematous base, frequently preceded or accompanied by neuropathic pain. The lack of lesions crossing the midline is a key diagnostic feature.^2^, ^11^, ^43^

Immunocompromised individuals and patients with disseminated disease may exhibit atypical or ambiguous presentations that resemble other vesiculobullous conditions.^23^, ^44^, ^61^ Relevant differential diagnoses include herpes simplex virus infections, impetigo, allergic contact dermatitis, bullous drug reactions, and autoimmune blistering disorders.^2^, ^11^, ^35^ A careful evaluation of the lesion distribution, morphology, temporal evolution, and pain characteristics helps delineate VZV from these mimickers.^33^, ^35^

Additional differentials for varicella include Mpox and, historically, smallpox. Although smallpox was eradicated in 1980, this comparison is clinically instructive.^35^ Mpox lesions are typically monomorphic and evolve more slowly, whereas varicella lesions are asynchronous at multiple developmental stages.^35^ Hand-foot-and-mouth disease may also resemble early varicella; however, its vesicles are confined predominantly to the hands, feet, and oral mucosa, with no tendency toward diffuse involvement.^35^

Other entities that may enter the differential diagnosis (particularly in atypical, severe, or disseminated presentations) include disseminated herpes simplex infection, *pityriasis lichenoides et varioliformis acuta*, rickettsial infections, drug eruptions, arthropod reactions, and scabies.^2^, ^33^, ^35^ A systematic approach that integrates morphology, distribution, symptom patterns, and host immune status enhances diagnostic accuracy and guides early therapeutic intervention.

### Laboratory and molecular tests

Laboratory confirmation is recommended when the clinical diagnosis is uncertain or in high-risk settings such as pregnancy or immunosuppression.^62^, ^63^ Polymerase Chain Reaction (PCR) testing, which detects VZV DNA in vesicular fluid, lesion crusts, or tissue biopsy specimens, is the most sensitive and specific method.^62^ PCR can differentiate between wild-type and vaccine strains and has largely replaced viral culture in clinical practice because of its rapid turnaround and higher sensitivity.^63^

Direct Fluorescent Antibody (DFA) testing remains a useful rapid diagnostic option when PCR is unavailable, although with lower sensitivity.^62^, ^64^, ^65^ The Tzanck smear, once widely used, can reveal multinucleated giant cells but cannot distinguish VZV from herpes simplex infection and is now of mainly historical interest.^66^, ^67^

Serological testing can demonstrate prior exposure or immune status by detecting VZV-specific IgG; however, it is not useful for diagnosing acute herpes zoster.^62^, ^64^, ^65^ Rising IgM titers may indicate recent infection; however, false negatives are frequent in reactivation cases.^62^, ^64^, ^65^

### Histopathology

**Figure 7:**
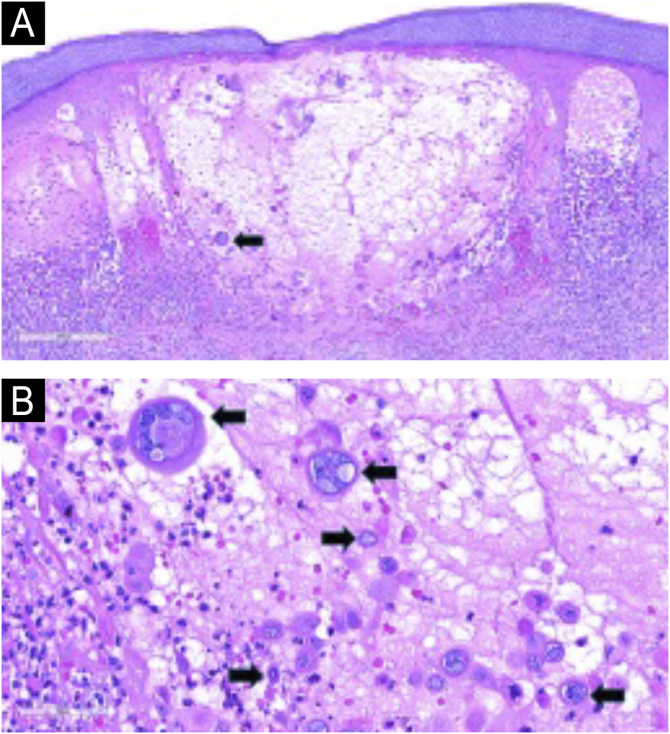
Skin histological section showing an intraepidermal vesicle with acantholysis, ballooning, and reticular degeneration in the epidermis. Note the multinucleated keratinocytes (arrow) and lymphocytic inflammatory infiltrate in the superficial dermis (Hematoxilyn & eosin, ×85) (A) Higher magnification demonstrating viral cytopathic changes. Multinucleated keratinocytes, nuclear molding, and peripheral chromatin margination ('ground glass' appearance) were observed (arrows) (Hematoxilyn & eosin, ×400) (B). Courtesy: Thiago Jeunon, MD.

Skin biopsy is rarely required but can be valuable in atypical, severe, and disseminated presentations. Histopathological examination typically demonstrates intraepidermal vesiculation with multinucleated keratinocytes, acantholysis, and eosinophilic nuclear inclusion bodies (Fig. 7).^68^ A superficial perivascular lymphocytic infiltrate, sometimes with scattered eosinophils, is also characteristic.^68^

The microscopic appearance of varicella closely mirrors that of herpes simplex virus infection, with both displaying multinucleated keratinocytes with molded nuclei and marginated chromatin.^68^ Because routine histology cannot reliably distinguish between the two, confirmatory studies are often necessary when morphology is ambiguous.^68^ Immunohistochemistry using VZV-specific antibodies or *in situ* hybridization can accurately identify viral DNA and resolve cases in which clinical or histological overlap complicates the diagnosis.^64^, ^65^, ^68^

### Diagnostic pitfalls in immunosuppressed and older adults

In immunocompromised individuals (such as patients with hematologic malignancies, organ transplants, or those undergoing immunomodulatory therapy), VZV infection may present without the classic vesicular eruption.^19^, ^20^, ^44^, ^57^ Lesions can be necrotic or hemorrhagic and can mimic bacterial or autoimmune blistering diseases.^35^ Disseminated zoster may develop without clear dermatomal limitation, and pain can be absent or minimal, leading to misdiagnosis as a drug reaction or vasculitis.^23^, ^44^, ^57^

Older adults may exhibit similar diagnostic challenges, particularly when neuralgia precedes the rash or when the eruption remains localized and subtle.^12^, ^69^ Misattributing this pain to musculoskeletal or neuropathic pain can delay antiviral treatment initiation and increase the risk of postherpetic neuralgia.^12^, ^26^, ^69^ Dermatologists should maintain a high suspicion in older adults or immunocompromised patients presenting with localized neuropathic pain or atypical vesiculobullous lesions.

Early PCR testing of lesion material or biopsy for VZV detection is recommended in ambiguous presentations, as prompt confirmation allows for immediate antiviral therapy and prevents dissemination.^64^, ^65^

## Treatment and prevention

Effective management of VZV infection aims to alleviate symptoms, accelerate lesion healing, and prevent complications, such as postherpetic neuralgia and secondary bacterial infection. General care and early antiviral therapy remain the cornerstones of treatment, complemented by appropriate pain control and preventive vaccination.

### General care

Supportive care remains essential for both varicella and herpes zoster, complementing antiviral therapy when indicated. Adequate hydration should be encouraged, particularly in patients with high fever, extensive lesions, or oral discomfort that may limit fluid intake.^33^, ^70^ Rest during the acute phase helps reduce systemic symptoms and may lower the risk of complications.^36^, ^49^, ^70^

Acetaminophen or ibuprofen is appropriate for fever and pain control across age groups. Acetylsalicylic acid is contraindicated in children and adolescents because of its association with Reye's syndrome.^33^, ^35^ Pruritus can be managed with topical agents such as calamine lotion, oatmeal baths, cold compresses, and regular emollient use, along with oral antihistamines when needed.^70^ To minimize excoriation and secondary bacterial infection, patients should be advised to keep their nails short and avoid scratching.^33^, ^35^

Preventing secondary infections is particularly important.^9^ Gentle cleansing of the affected area and application of antiseptic solutions, such as povidone-iodine, chlorhexidine, or boric acid, can reduce bacterial colonization.^37^, ^49^ When signs of impetiginization arise, topical antibiotics may be used as adjunctive therapy while antiviral treatment proceeds.^9^, ^49^, ^71^

### Antiviral therapy

Oral antiviral therapy should be considered for patients with a substantial likelihood of severe disease to shorten the clinical course of varicella, reduce symptom intensity, and limit the risk of complications, oral antiviral therapy should be considered for patients with a substantial likelihood of severe disease.^33^, ^35^ This group includes unvaccinated individuals aged ≥13-years, secondary household cases (which often manifest more intense presentations due to higher inoculum), patients with chronic dermatologic or pulmonary conditions, children receiving prolonged oral or inhaled corticosteroids, individuals with long-term acetylsalicylic acid exposure, and pregnancy.^33^, ^35^ In contrast, healthy children typically do not benefit from antiviral therapy, as varicella in this population follows a self-limited course, and treatment provides only modest improvements.^33^, ^35^

Intravenous antiviral therapy is recommended for immunocompromised individuals, such as those with malignant neoplasms, HIV infection, or ongoing immunosuppressive regimens.^35^, ^49^, ^63^ In varicella, timing is essential: treatment started within the first twenty-four hours after rash onset consistently yields the greatest reduction in viral replication and lesion progression.^33^, ^35^

For herpes zoster, systemic antiviral therapy is recommended for all patients.^9^, ^72^, ^73^ Initiation within seventy-two hours of rash onset provides clearer clinical benefits, notably by limiting the formation of new lesions and reducing acute neuritic pain.^63^, ^74^ Most patients can be effectively managed with oral agents; intravenous therapy becomes necessary when dissemination, ocular involvement, or significant immunosuppression is present.^47^, ^49^, ^63^

Acyclovir, valacyclovir, and famciclovir remain the principal therapeutic agents (Table 1). Valacyclovir and famciclovir provide superior bioavailability and simpler dosing schedules compared with acyclovir, which often facilitates adherence and supports a more consistent therapeutic response.^58^, ^59^, ^73^, ^75^^,^^76^

**Table 1:** Antiviral therapy for Varicella-Zoster virus.

Drug	Dose (adults)	Dosing Interval	Typical Duration
Acyclovir	800 mg orally	Every 4 hours (5×/day)	7‒10 days
	10 mg/kg intravenous	Every 8 hours (3×/day)	7‒10 days
Valacyclovir	1,000 mg orally	Every 8 hours (3×/day)	7 days
Famciclovir	500 mg orally	Every 8 hours (3×/day)	7 days

Note: Dose adjustment is required for all nucleoside analogues in patients with impaired renal function.

Adjunctive corticosteroids may be considered for herpes zoster in selected immunocompetent adults with severe pain or extensive rashes, provided that antiviral therapy is administered concomitantly.^49^, ^72^ Their use remains controversial because they do not prevent postherpetic neuralgia and may pose risks to older or frail individuals.^47^ Topical antivirals have limited efficacy and are not recommended for use.^63^

### Pain management

Pain control is central to the management of herpes zoster and often requires a multimodal approach. During the acute phase, nonsteroidal anti-inflammatory drugs, analgesics, or short courses of opioids can be used to treat nociceptive pain.^77^ Neuropathic pain agents, such as gabapentin, pregabalin, or tricyclic antidepressants, should be introduced early if neuralgia develops, and even in the acute phase.^24^, ^77^, ^78^ Topical lidocaine patches and capsaicin creams may provide additional relief.^9^

Persistent pain lasting beyond 90-days is defined as PHN.^24^, ^77^, ^78^ Management may require higher doses of gabapentinoids, combination therapy, or referral to pain specialists.^24^, ^79^, ^80^ Preventive strategies, particularly early antiviral therapy and vaccination, are more effective than late-stage treatment.^47^

### Vaccination and prevention

Vaccination has transformed the epidemiology of VZV infections, markedly reducing the incidence of varicella and herpes zoster.^81^, ^82^

For the prevention of varicella, routine childhood immunization is based on a two-dose schedule with a live-attenuated varicella vaccine administered at 15-months (as part of the tetravalent formulation) and at 4-years of age, conferring durable protection.^83^ Although breakthrough infections may occur, they are typically mild. In a small proportion of vaccinated individuals, the attenuated vaccine strain may later reactivate and manifest as herpes zoster.^31^

Recent updates in immunization policies have refined the recommendations regarding combined formulations. In 2025, the Advisory Committee on Immunization Practices of the Centers for Disease Control and Prevention (CDC) recommended the suspension of the combined Measles, Mumps, Rubella, and Varicella (MMRV) vaccine due to safety concerns, favoring the separate administration of MMR and varicella vaccines in young children.^84^ Divergent positions remain among professional societies, and national schedules may continue to evolve.^85^

In contrast, the prevention of herpes zoster has undergone a definitive shift. Although a live-attenuated zoster vaccine (Zostavax®) was previously available, it is no longer recommended by the CDC and was discontinued in the United States in 2020.^86^, ^87^ In Europe, its marketing authorization was formally withdrawn by the European Commission in June 2025 at the request of the manufacturer.^86^, ^87^ Consequently, live-attenuated zoster vaccines no longer play a role in current prevention strategies against VZV.

The recombinant subunit vaccine (Shingrix®) is now the global standard of care for herpes zoster prevention.^82^ It is composed of VZV glycoprotein E combined with the AS01B adjuvant and induces robust and sustained immune responses, including in older adults and immunocompromised populations.^4^, ^27^ The vaccine is administered in two intramuscular doses given 2–6 months apart and is recommended even for individuals with a prior history of herpes zoster.^88^ Vaccination may be initiated six months after an acute episode, with earlier administration acceptable when clinically appropriate.^88^ Efficacy consistently exceeds 90% against herpes zoster and postherpetic neuralgia.^29^, ^30^, ^88^, ^89^ Local reactogenicity is common but transient.^89^ In comparison, live-attenuated zoster vaccines confer lower and less durable protection and are contraindicated in immunocompromised individuals.^90^, ^91^

## Post-exposure prophylaxis

The administration of varicella-zoster immunoglobulin (125 U/10 kg, up to a maximum of 625 U), given intramuscularly within 96 h of exposure, is recommended for post-exposure prophylaxis in immunocompromised non-immune adults, pregnant women, and high-risk neonates.^33^, ^35^ This passive immunization provides temporary protection, with suppression of clinical disease lasting approximately three weeks.^33^, ^35^ Intravenous immunoglobulin, administered at doses ≥0.4 g/kg and containing high titers of anti-varicella-zoster virus IgG, represents an alternative option in selected settings.^35^

Prophylaxis with oral acyclovir at a standard varicella dose may also be considered for 1-week, starting 7–10 days after exposure.^35^ Furthermore, post-exposure vaccination with a live attenuated virus vaccine can prevent or mitigate the clinical picture when administered within 72–120 hours and is indicated for non-immune individuals aged one year or older, provided they are immunocompetent and eligible for immunization.^35^

## Practical guidance and conclusions

VZV remains a significant cause of dermatologic morbidity, bridging fundamental virology, immunology, and clinical practice. Its two clinical faces (varicella as a primary infection and herpes zoster as reactivation) represent distinct biological expressions of the same pathogen, each shaped by host immunity and age-related vulnerability. For dermatologists, understanding this continuum provides a conceptual framework for accurate diagnosis and evidence-based therapies.

Advances in molecular diagnostics and antiviral pharmacotherapy have transformed clinical outcomes; however, delayed recognition and underdiagnosis persist, particularly in atypical or immunocompromised presentations. The advent of recombinant subunit vaccination marks a paradigm shift toward prevention through durable immunologic control rather than reactive management. As this preventive model matures, the dermatologist’s role expands beyond treatment to encompass patient counseling, vaccine advocacy, and the integration of immunization strategies into everyday practice.

Ultimately, the management of VZV infection exemplifies the convergence of basic science and applied dermatology. Through sustained clinician awareness, rapid therapeutic intervention, and widespread vaccination, the burden of VZV and its complications can be substantially reduced in the future.

## ORCID IDs

Maria Paula Barbieri D’Elia: 0000-0003-1524-721X

Carla Riama Lopes de Pádua Moura: 0000-0002-5092-2180

Rafael de Deus Moura: 0000-0002-2260-1179

Juliana de Sá Pires Carvalho: 0009-0009-7661-0984

Henrique Pott: 0000-0003-3126-2946

## Research data availability

The entire dataset supporting the results of this study was published in this article.

## Financial support

This research received no specific grants from funding agencies in the public, commercial, or not-for-profit sectors. However, HP was supported by a fellowship from the Coordenação de Aperfeiçoamento de Pessoal de Nível Superior ‒ Brasil (CAPES) ‒ Finance Code 001.

## Authors' contributions

Maria Paula Barbieri D'Elia: Design and planning of the study; collection of data; drafting and editing of the manuscript and critical review of important intellectual content; data collection, analysis, and interpretation of data; effective participation in research orientation; critical review of the literature; approval of the final version of the manuscript.

Carla Riama Lopes de Pádua Moura: Design and planning of the study; collection of data; drafting and editing of the manuscript and critical review of important intellectual content; collection, analysis, and interpretation of data; effective participation in research orientation; critical review of the literature; approval of the final version of the manuscript.

Rafael de Deus Moura: Collection of data; drafting and editing of the manuscript and critical review of important intellectual content; collection, analysis, and interpretation of data; critical review of the literature; approval of the final version of the manuscript.

Juliana de Sá Pires Carvalho: Collection of data; drafting and editing of the manuscript; critical review of the literature; collection, analysis, and interpretation of data; approval of the final version of the manuscript.

Henrique Pott: Design and planning of the study; collection of data; drafting and editing of the manuscript and critical review of important intellectual content; collection, analysis, and interpretation of data; effective participation in research orientation; critical review of the literature; approval of the final version of the manuscript.

## Conflicts of interest

MPBD, CRLPM, RDM, and JSPC declare no conflicts of interest. HP declares no additional conflicts of interest beyond the aforementioned institutional support. Henrique Pott received funding from the Coordenação de Aperfeiçoamento de Pessoal de Nível Superior - Brasil (CAPES) ‒ Finance Code 001. The remaining authors declare that they have no conflicts of interest.
